# The patient perspective on use of Omalizumab in the in-hospital setting

**DOI:** 10.1007/s11845-025-03978-5

**Published:** 2025-06-25

**Authors:** Lara Dungan, Fiona Little, Niamh O’Connor, Fionnuala Cox

**Affiliations:** Department of Clinical Immunology, Beaumont Hospital, Beaumont Road, Dublin 9, Dublin, Ireland

**Keywords:** Chronic spontaneous urticaria, Home therapy, Omalizumab, Quality of life

## Abstract

**Background:**

Omalizumab is approved for the treatment of chronic spontaneous urticaria (CSU), allergic asthma, and chronic rhinosinusitis with nasal polyps. While self-administration is licensed in Ireland, reimbursement restrictions require hospital-based delivery, placing significant burdens on patients and healthcare services.

**Aims:**

To evaluate patient perspectives on hospital-based administration of Omalizumab, and assess the practical, financial, and environmental implications of current practices, alongside interest in transitioning to home-based therapy.

**Methods:**

A cross-sectional survey was conducted among patients receiving Omalizumab in a tertiary referral hospital between December 2024 and January 2025. Eligible participants (n=49) completed a 20- question anonymous questionnaire exploring demographics, treatment burden, and attitudes toward home therapy. Cost data were obtained from institutional finance records.

**Results:**

Among 49 respondents (98% response rate), 46.9% reported personal costs of €11–€50 per hospital visit, with some incurring over €1,300 annually. Over one quarter (26.5%) missed more than 11 workdays per year due to treatment. Most travelled by private vehicle, generating an estimated 2.1 tonnes of CO₂ annually. A majority (77.5%) would prefer home therapy, citing convenience, flexibility, and reduced financial burden. Annual institutional costs for hospital-based administration of Omalizumab exceeded €1 million, excluding drug expenditure.

**Conclusions:**

Hospital-based administration of Omalizumab imposes significant patient and system-level costs. These findings support a transition to reimbursed self-administration at home, which may improve patient satisfaction, reduce absenteeism, minimise environmental impact, and achieve considerable healthcare savings.

**Supplementary Information:**

The online version contains supplementary material available at 10.1007/s11845-025-03978-5.

## Introduction

Omalizumab is a monoclonal anti-immunoglobulin E (IgE) antibody used to treat allergic asthma, CSU, chronic rhinosinusitis with nasal polyps, and IgE-mediated food allergy [1]. Approved for CSU treatment in over 80 countries, Omalizumab, manufactured in a pre-filled syringe, is licenced for self-administration by both the European Medicines Agency (EMA) and the Food and Drug Administration (FDA) [2]. Omalizumab has been licensed for self-administration in Ireland since 2018 [3], however reimbursement policies do not support home-based administration in Ireland, which is in stark contrast to most other health services and jurisdiction [4, 5]. In Ireland Omalizumab administration requires admission to a day ward or ambulatory care unit. The standard starting dose interval for the treatment of CSU is 4 weekly [6]. This study investigates the patient perspective on in-hospital Omalizumab administration, considering the economic, environmental, and practical implications, as well as the potential for transitioning to home-based therapy.

## Methods

This cross-sectional study included patients attending a large tertiary hospital for Omalizumab administration during a four-week period (December 2024-January 2025). The centre is one of only three Clinical Immunology centres nationally. Eligible patients were > 16 year of age and established on Omalizumab treatment. Participants were approached by nursing staff and provided with an information leaflet. Written informed consent was obtained prior to completing a 20-question anonymous questionnaire, which covered demographics, treatment history, attitudes toward therapy effectiveness, and preferences for transitioning to home therapy (supplementary material, Fig. 1). This survey was partially based on a questionnaire of CSU patients receiving Omalizumab from a previous study [7]. Ethical approval was granted by Beaumont Hospital’s Medical Research Ethics Committee.

A costing analysis was conducted to estimate the financial impact of hospital-based Omalizumab administration, including direct costs for personnel, facilities, and logistical support. The financial data were provided by the hospital’s finance department.

## Results

Fifty patients were approached, and 49 completed the survey (98% response rate). The mean age of participants was 43.6 years (range of 17 to 69 years), and the majority (53.1%) were female.

Nearly half (46.9%; 23/49) of respondents incurred personal costs between €11 and €50 per hospital visit, with some patients spending over €1,300 annually on treatment-related costs. Additionally, 26.5% (13/49) missed more than 11 days of work annually solely due to Omalizumab-related hospital visits. The majority of patients (85.7%; 35/49) reported a potentially substantive time saving, up to 5 h for some (14.2%; 7/49), if at home self-administration of Omalizumab was supported.

Most patients (85.7%; 42/49) travelled by private petrol or diesel fuelled vehicle, with 73.5% (36/49) traveling over 10 km for each visit. Nine patients had round trips exceeding 100 km, equating to an excess of 10,622 km driven, resulting in at least 2.1 tonnes of CO_2_ emissions per year.

A majority (77.5%; 38/49) of patients expressed interest in switching to home therapy, citing benefits such as cost savings, time savings, greater flexibility, and fewer hospital visits. Concerns about self-injection were the primary barrier for the remaining 22.5% (11/49), with many fearing incorrect injection techniques, missed doses, and side effects. Most concerns could be alleviated with a patient-focused training programs for self-administration of injectable medication as well as education regarding scheduling of medication and known side-effects.

The cost for a single Omalizumab day ward visit in 2023 was €1,198, with an estimated €1,051,844 spent annually for administering Omalizumab across 878 visits. This excludes the cost of the medication itself.

## Discussion

Our findings show a considerable appetite for transitioning to home Omalizumab therapy among patients – a practice that is licensed for patients with CSU, but not yet supported for reimbursement at a community level. Home-based self-administration aligns with trends observed in other countries where this is standard practice.

There is scant information in the literature about the patient perspective when it comes to administration of Omalizumab at home versus in hospital. One German study [8] demonstrated 44.7% of patients with severe asthma favoured home administration. In our cohort the figure is 77.5% (38/49) in favour of home therapy. This disparity may be reflective of differences in the underlying disease being treated or reflective of an emerging preference for home and community based care in this post COVID19 pandemic era. In addition, the literature is sparse when it comes to research on how patients have adapted after commencing self-administration with biologics.

Our data indicate a strong preference in our patient cohort for transitioning to home therapy. There is a potential superimposed psychological burden of in-hospital administration of Omalizumab in this patient group associated with time, employment and financial stressors by remaining in the in hospital setting. It is acknowledged that psychological stress can exacerbate and re-ignite dormant CSU symptoms [9], thus transition to home-based treatment may in itself be a therapeutic intervention in this patient population.

Not only does it cost our institution in excess of one million euro per year to facilitate admission, in a service with long waiting lists, this could be considered a misallocation of resources. Our data demonstrate that transition to home therapy is a logical, safe and cost saving solution allowing greater patient autonomy, flexibility and satisfaction. The potential cost and time savings, along with reduced environmental impact, are compelling reasons to consider home-based Omalizumab therapy. Moreover, the high personal costs, absenteeism and travel burdens associated with in-hospital treatment highlight the need for review of current practice. Our study suggests that implementing home therapy could not only improve patient autonomy and satisfaction but also lead to significant cost reductions for healthcare systems.

## Supplementary Information

Below is the link to the electronic supplementary material.

ESM 1 (DOCX 21.2 KB)

## Data Availability

Not applicable.
