# Supplementary material for: The patient perspective on use of Omalizumab in the in-hospital setting
Source: Ir J Med Sci. 2025 Jun 25;194(3):793–5. doi: 10.1007/s11845-025-03978-5 (PMC12276100; doi:10.1007/s11845-025-03978-5)
Supplement: Supplementary file 1 — (DOCX 21.2 KB) [file 11845_2025_3978_MOESM1_ESM.docx]

Supplementary Materials

Fig1 – Patient Questionnaire

**Patient Questionnaire for Omalizumab (Xolair) home use**

1. Questionnaire respondent
   - I am the patient
   - I am filling out this questionnaire on behalf of the patient
     1. What is your relationship to the patient? __________________

**Please answer the remainder of the questions in relation to the patient, unless otherwise specified.**

1. Gender
   - Male
   - Female
   - Prefer not to answer
2. Age: ______ years
3. For what condition do you receive Omalizumab?
   - Recurrent unprovoked hives (ie. Chronic Spontaneous Urticaria)
   - Recurrent unprovoked swellings (ie. Chronic Spontaneous Angioedema)
   - Recurrent unprovoked hives **and** swellings (Chronic Spontaneous Urticaria-Angioedema)
   - Nasal polyps
   - Other: ___________________________________________________
   - Don’t know
4. How long approximately have you been receiving Omalizumab therapy?
   - Less than 1 year
   - 1 – 4 years
   - 5 – 9 years
5. For how long did you have **symptoms** before starting Omalizumab therapy?
   - Less than 1 year
   - 1 – 4 years
   - 5 – 9 years
   - Greater than 10 years
6. Generally speaking, how satisfied or dissatisfied are you with the way Omalizumab manages your symptoms?
   - Extremely satisfied
   - Satisfied
   - Somewhat satisfied
   - Dissatisfied
   - Extremely dissatisfied
7. In what dosing intervals do you currently receive Omalizumab?
   - 3 weeks
   - 4 weeks
   - 6 weeks
   - Other: ___________________
8. What dose of Omalizumab do you currently receive at each visit?
   - 300mg (2 syringes)
   - Other: _______syringes, or ________mg
9. How flexible would you describe the Omalizumab therapy **in the hospital setting**?
   - Not very flexible
   - Flexible
   - Highly flexible
10. What are your personal expenses for each Omalizumab administration **in the hospital setting**? (public transport costs, private transport – fuel, parking etc)
    - Less than €10
    - €11 - €20
    - €20 - €50
    - Over €50
11. How many days per year do you lose, due to the time taken for your Omalizumab administration **in the hospital setting**? (eg. taking leave from work for dayward attendances)
    - <1 days
    - 1-10 days
    - 11-20 days
    - >20 days
12. How far do you have to travel to get to hospital for administration of your Omalizumab injection? (please include your round trip distance – both your trips to and from the hospital)
    - Less than 10 km
    - 10-50km
    - 50-100km
    - Over 100km
13. What mode of transportation do you generally use to travel to your appointment for Omalizumab administration?
    - Walk
    - Cycle
    - Public transport (bus, train etc)
    - Private motor car (petrol/diesel)
    - Private motor car (electric)
    - Other: ___________________
14. Currently, Omalizumab is not available for home administration (by patient or care-giver/family member) in Ireland as it is not funded in the community. If it were to become available, and training was completed in the hospital setting, would you be happy to switch to self-injected home therapy? *(Please give a reason for your choice)*
    - Yes, I would be happy to self-administer Omalizumab at home.
    - No, I would prefer to remain with hospital-based treatment.

Please comment below:

1. For each dose, approximately how many hours would you save by administering Omalizumab at home?
   - Less than 1 hour
   - 1-5 hours
   - Over 5 hours

**Please indicate the extent to which you agree with the following statements.**

*Please answer the questions by indicating to what extent you agree with the statements. If you have no experience regarding a particular subject, please answer ‘not applicable’.*

General questions

|  | ***Strongly agree*** | ***Partially agree*** | ***Neutral*** | ***Partially disagree*** | ***Strongly disagree*** | *Not applicable* |
| --- | --- | --- | --- | --- | --- | --- |
| **17. I am in favour of administering Omalizumab at home** |  |  |  |  |  |  |
| **18. For me, administering Omalizumab at home would lead to:** |  |  |  |  |  |  |
| 1. Cost savings |  |  |  |  |  |  |
| 1. Time savings |  |  |  |  |  |  |
| 1. Greater flexibility in my daily life |  |  |  |  |  |  |
| 1. Less hospital visits |  |  |  |  |  |  |
| 1. Better quality of life |  |  |  |  |  |  |
| 1. Less risk of exposing myself to hospital borne infections |  |  |  |  |  |  |

Questions relating to patient concerns

|  | ***Strongly agree*** | ***Partially agree*** | ***Neutral*** | ***Partially disagree*** | ***Strongly disagree*** | *Not applicable* |
| --- | --- | --- | --- | --- | --- | --- |
| **19. I worry about the following in relation to self-injecting at home:** |  |  |  |  |  |  |
| 1. Injecting myself incorrectly |  |  |  |  |  |  |
| 1. Forgetting to take a dose |  |  |  |  |  |  |
| 1. Developing a side-effect or reaction to the medicine |  |  |  |  |  |  |

**20. If you have any other comments of suggestions please let us know?**

Please comment below:

**Thank you for completing this questionnaire!**
